# Reweighting a Swedish health questionnaire survey using extensive population register and self-reported data for assessing and improving the validity of longitudinal associations

**DOI:** 10.1371/journal.pone.0253969

**Published:** 2021-07-01

**Authors:** Anton Nilsson, Carl Bonander, Ulf Strömberg, Catarina Canivet, Per-Olof Östergren, Jonas Björk

**Affiliations:** 1 EPI@LUND (Epidemiology, Population Studies and Infrastructures), Department of Laboratory Medicine, Lund University, Lund, Sweden; 2 School of Public Health and Community Medicine, Institute of Medicine, Sahlgrenska Academy, University of Gothenburg, Gothenburg, Sweden; 3 Department of Research and Development, Region Halland, Halmstad, Sweden; 4 Social Medicine and Global Health, Lund University, Lund, Sweden; 5 Clinical Studies Sweden, Forum South, Skåne University Hospital, Lund, Sweden; Ruhr University Bochum, GERMANY

## Abstract

**Background:**

In cohorts with voluntary participation, participants may not be representative of the underlying population, leading to distorted estimates. If the relevant sources of selective participation are observed, it is however possible to restore the representativeness by reweighting the sample to resemble the target population. So far, few studies in epidemiology have applied reweighting based on extensive register data on socio-demographics and disease history, or with self-reported data on health and health-related behaviors.

**Methods:**

We examined selective participation at baseline and the first two follow-ups of the Scania Public Health Cohort (SPHC), a survey conducted in Southern Sweden in 1999/2000 (baseline survey; n = 13,581 participants, 58% participation rate), 2005 (first follow-up, n = 10,471), and 2010 (second follow-up; n = 9,026). Survey participants were reweighted to resemble the underlying population with respect to a broad range of socio-demographic, disease, and health-related characteristics, and we assessed how selective participation impacted the validity of associations between self-reported overall health and dimensions of socio-demographics and health.

**Results:**

Participants in the baseline and follow-up surveys were healthier and more likely to be female, born in Sweden, middle-aged, and have higher socioeconomic status. However, the differences were not very large. In turn, reweighting the samples to match the target population had generally small or moderate impacts on associations. Most examined regression coefficients changed by less than 20%, with virtually no changes in the directions of the effects.

**Conclusion:**

Overall, selective participation with respect to the observed factors was not strong enough to substantially alter the associations with self-assessed health. These results are consistent with an interpretation that SPHC has high validity, perhaps reflective of a relatively high participation rate. Since validity must be determined on a case-by-case basis, however, researchers should apply the same method to other health cohorts to assess and potentially improve the validity.

## Introduction

Cohort studies used in epidemiology and medicine are usually recruited on a voluntary basis and are as such made up of limited and potentially selective groups of individuals. In recent decades, participation in cohorts has dropped substantially from about 80% to 30–40% [1], perhaps making participants increasingly less representative of the underlying populations. Lack of representativeness due to selective participation is concerning, as it can lead the researcher to draw conclusions about prevalences, incidences, and associations that do not generalize to the population as a whole [2–4], an issue referred to as *selection bias* [5]. In the case of associations, selection bias can reflect that associations obtained among cohort participants lack causal interpretation (*collider stratification bias*) [6, 7] or that causal effects are inherently different across different groups of individuals (*effect heterogeneity*) [8, 9].

Lack of representativeness is a relatively well-recognized problem in cohort studies, but few previous studies in epidemiology have made attempts to correct for it. As demonstrated by a recent and growing theoretical literature, however, there is a method to restore representativeness that can easily be applied, at least to the extent that relevant background variables are observed for both the participants and for the underlying background population [3, 10–13]. The method, sometimes referred to as *inverse probability of participation weighting* (IPPW), involves the estimation of individual participation probabilities, followed by analyses conducted on a reweighted participant sample. Previous applications of the method to cohort studies have yielded mixed results, although it has appeared that measures of association may often be relatively unaffected by reweighting [14–16].

The Scania Public Health Cohort (SPHC) is a general population study conducted in Scania County, Southern Sweden [17, 18]. In late 1999 and early 2000, a questionnaire about health and social circumstances was sent out to a geographically stratified sample of 23,437 persons born 1919–1981 and living in the county. The questionnaire was returned by 58% of these individuals (n = 13,581). All respondents of the baseline survey who were alive and resided in Scania were then sent similar follow-up surveys in 2005, 2010, and 2016.

The issue of selective participation in SPHC has previously been investigated [17, 18]. At baseline, young individuals, men, individuals with low education, and immigrants were found to be underrepresented. Overall, however, underrepresentation was not found to be substantial. Similarly, in the first follow-up, young individuals, men, immigrants, and low-educated, as well as smokers and individuals with bad self-assessed health were found to be somewhat less likely to participate. Neither of these studies made any effort to improve the validity of associations by applying IPPW or similar methods.

In the present study, we examined determinants of selective participation (“selection”) in SPHC and the consequences of reweighting the participants to reduce selection bias. We considered selection with respect to an extensive set of background data, including both socio-demographics and disease history from national registers, and self-assessed health, overweight, smoking behavior, and physical activity from previous rounds of SPHC. Selection into both baseline and the first two follow-ups was considered (data from the third was not available to us), and the surveys were reweighted with respect to the background data for improved representativeness. To our knowledge, no previous study has been able to reweight a health cohort with respect to such extensive data. We examined associations between self-assessed general health and numerous socio-demographic, disease, and health-related characteristics, contrasting unweighted and reweighted results.

## Materials and methods

### Data

Three classes of data were used for this study: 1) the SPHC study held at Lund University, 2) register data on socio-demographic variables from Statistics Sweden, and 3) register data on hospital visits from the National Board of Health and Welfare. The three datasets were linked together by Statistics Sweden, using personal identifiers. Two separate merged datasets were delivered to us: one on the SPHC participants and one on a representative sample from the target population. The sample from the target population included 60,000 inhabitants of Scania born 1919–1981 and was geographically stratified in a way such as to correspond to the stratified sample invited to SPHC.

The sample of SPHC participants included the 13,581 individuals who had returned the baseline questionnaire in 1999/2000. Variables available reflected the answers the individual had provided in the baseline questionnaire as well as answers in the 2005 and 2010 follow-up questionnaires in case the individual had participated and responded to any of these. Among the 12,629 baseline participants who still were alive and lived in Scania by 2005, 10,471 (83%) participated in the 2005 follow-up, and among the 11,737 baseline participants who were still alive and lived in Scania by 2010, 9,026 (77%) participated in the 2010 follow-up.

Questions included in SPHC pertained to self-assessed health, use of medications, anthropometry, health-related behaviors, social participation, sickness absence, working conditions, and economic circumstances. We exploited data on overall self-assessed health at baseline and in the two follow-ups, and BMI, leisure time physical activity, and smoking at baseline and in the first follow-up. Self-assessed health was reported by the individual on a five-step scale (very good, good, fair, poor, and very poor). Physical activity was reported on a four-step scale: 1) “Sedentary: read, do handwork, watch TV, go to the movies, and similar sedentary activities,” 2) “Moderate exercise: walk, bike, or other physical activity for at least four hours per week,” 3) “Regular exercise: running, swimming, tennis, badminton, gymnastics or similar,” 4) “Hard exercise: Engage in hard exercise or competition, for example running, orientation, skiing, swimming, soccer, handball regularly and several times per week.” Smoking was reported in three categories (“smoking, daily,” “smoking, not daily,” and “not smoking”). BMI was obtained via the individual’s self-reports of height and weight, and we used an indicator for “overweight,” corresponding to a BMI of at least 25.

Individuals who had not provided answers on overall self-assessed health, physical activity, smoking, height, or weight were removed, reducing the baseline sample by 8% to 12,432 individuals, the 2005 follow-up by 6% to 9,836 individuals, and the 2010 follow-up by 5% to 8,565 individuals. Moreover, since analyses of outcomes in the 2005 and 2010 rounds required information from previous rounds of SPHC, attention was restricted to those who provided information on the above-mentioned variables in the previous rounds. This further reduced the 2005 participant sample by 5% to 9,315 individuals and the 2010 participant sample by 7% to 7,104 individuals.

We had access to register data from Statistics Sweden spanning 1990–2016. Demographic variables used were age, sex, country of birth (grouped), and civil status (dichotomized to married and not married). Socioeconomic variables used included educational attainment, employment status, and disposable income (total individual income after taxes).

Register data on hospitalizations from the National Board of Health and Welfare spanned the years 1987–2016. Inpatient hospital visits were available from 1987, whereas outpatient hospital visits were available from 2001. Data included dates of admission and discharge, and the diagnosis code for the main diagnosis associated with the visit, coded based on the International Classification of Diseases (ICD), version 9 or 10. We considered disease history with regard to the following diagnose groups: neoplasms (ICD codes 140-239/C00-D48), diabetes (250/E10-E14), mental and behavioral disorders (290-319/F00-F99), diseases of the circulatory system (390-459/I00-I99), diseases of the respiratory system (460-519/J00-J99), and diseases of the digestive system (520-579/K00-K93).

### Data analysis

We reweighted SPHC participants to match the characteristics of the sample of 60,000 individuals that was drawn from the target population. The applied procedure was similar to the standard IPPW method, but since data on participants and the target population were delivered as two separate datasets, we used a modified version, reweighting baseline participants with respect to estimated *odds* of participation rather than the probability; see [13] for an introduction to this modified approach. The modified method gives accurate results because odds of participation in a dataset that combines participants and individuals from (a random sample of) the full target population correspond to probabilities of actually participating [13]. We hence combined the two datasets and applied logistic regressions with baseline participation as the outcome and obtained our weights as the inverses of the predicted individual-specific odds from these regressions. Predictors included socio-demographic variables from 1999 and indicators for whether the individual had at least one hospital visit of different types in 1987–1999.

As our main interest was in longitudinal associations, we also had to reweight the 2005 and 2010 follow-ups. We here applied *double* and *triple* reweighting schemes, respectively, accounting for both selection into baseline and into the follow-ups. Specifically, for the 2005 follow-up, a total weight was obtained by multiplying the inverse of the predicted odds of participating at baseline by the inverse predicted probability of participating in the 2005 follow-up, given participation at baseline. The latter was obtained from a logistic regression applied to baseline participants, using socio-demographic variables from 2004, hospital visits from 1987–2004, and self-assessed variables from the baseline questionnaire as predictors. For the 2010 follow-up, this weight was further multiplied by the inverse of the predicted odds of participating in the 2010 follow-up, given participation in both the baseline survey and 2005 follow-up. The weight reflecting selection into the 2010 follow-up was based on socio-demographic variables from 2009, hospital visits from 1987–2009, and self-assessed variables from the 2005 questionnaire. A similar stepwise reweighting procedure has been used previously to deal with sequential dropouts [19].

The goal of the reweightings was to generalize associations to the target population. We did not exclude individuals who had died or migrated out of Scania after baseline when estimating the participation models, as doing so would have given rise to more selected samples, potentially leading to biased results. Instead, individuals not participating in follow-up surveys were counted as non-participants regardless of whether their non-participation was an active decision or caused by death or migration. For persons who had moved from Scania to another region of Sweden, we were still able to obtain the register data needed for the reweightings; in contrast, if a person had died or moved out of country, we used socio-demographic data from the last available year and hospital visits up until the last available year when estimating the probability of participating in the follow-up.

The fact that the predicted probabilities of baseline participation were represented by odds implied that values greater than one could sometimes occur. In practice, this issue was minor, however, and occurred only for 0.7% of participants and 0.02% of non-participants. In the calculations of weights, we rounded these participation probabilities down to one (keeping them above one or removing individuals with values above one made virtually no difference to the results, however).

Having reweighted the data on participants, we compared results from unweighted and weighted logistic regressions for associations with self-assessed overall health. Here, self-assessed health was dichotomized into “bad” (representing “poor,” “very poor,” or “fair” health on the underlying five-step scale) and “not bad” (representing “good” or “very good”). Overall, the explanatory variables were based on the same variables that were used to predict the probability of participation in the corresponding survey, but we used somewhat condensed sets of them in order to limit the amount of output. Country of birth was dichotomized into native-born and foreign-born. Physical activity was dichotomized into “sedentary” and “non-sedentary,” and smoking was dichotomized into “daily smoker” and “not daily smoker.” When examining associations with bad health at baseline, we added self-assessed variables from the baseline survey to the set of explanatory variables, as data from previous rounds were not available.

To display the ability of our participation models to distinguish between participants and non-participants, histograms of predicted participation probabilities were constructed; one for each round of the survey. A complicating issue was that non-participants in the baseline survey could not be directly identified in the data. Participation probabilities for this group were therefore obtained via the law of total probability, exploiting predicted participation probabilities in the target population as well as in the participant sample. Specifically, the law of total probability implies the following equation for the shares of individuals in different intervals with respect to participation probability:

P(a≤Q<b)=P(a≤Q<b|C=1)*P(C=1)+P(a≤Q<b|C=0)*P(C=0) (1)

Here, *Q* is an individual’s probability of being a baseline participant and *C* is actual baseline participation. *a* and *b* are bounds of an interval in the histogram. *Q* was obtained from a logistic regression applied to the combined sample of participants and the target population, as previously described. For each interval, the left-hand side of the equation was identified from the target population and the first factor on the right-hand side from the participant sample. The corresponding quantity for non-participants was then obtained by rewriting the equation:

$$P(a\leq Q<b|C=0)=\frac{P(a\leq Q<b)-P(a\leq Q<b|C=1)*P(C=1)}{P(C=0)}$$ (2)

Using Eq (2), a quasi-population of non-participants was constructed, which was used for the construction of the histogram of participation probabilities in the baseline survey.

The ability of the background variables to correctly classify individuals with respect to participation was examined by calculating AUC (area under the ROC curve). For the baseline cohort, we here exploited the quasi-population of non-participants described above.

Analyses were performed using Stata 15.1 (StataCorp).

### Ethics statements

The project has been approved by the Regional Ethics Review Board in Lund (Dnr: 1999/99, 2005/471, 2010/382, 2015/471, and 2017/846). All data used has been pseudonymized, i.e., names or official personal numbers have not been visible to researchers working with the data.

The survey was sent by mail to the respondents. About a week before they received the survey form, individuals were alerted of it by a short letter describing the purpose of the study and informing that participation was voluntary. Attached to the survey form, there was further information concerning the study, stating that survey information would be linked to register information (on socioeconomics, healthcare visits, and similar) and once again they informing that participation was voluntary and that they agreed to participate by submitting a completed form. Moreover, individuals were informed that they could contact the researchers at any time and request their information to be deleted.

For individuals only appearing in the national register data and not in the survey, informed consent was not obtained. As stated by the Swedish law of Research Ethics, national register data can be used for research purposes without informed consent if approval has been obtained from an ethics board. The Regional Ethics Review Board granted us approval to use national register data without informing individuals appearing in the data and without their consent under the condition that information about the project was published at www.lupop.lu.se and that individuals were given the opportunity to be removed from the study by contacting co-author J.B.

## Results

**Fig 1:**
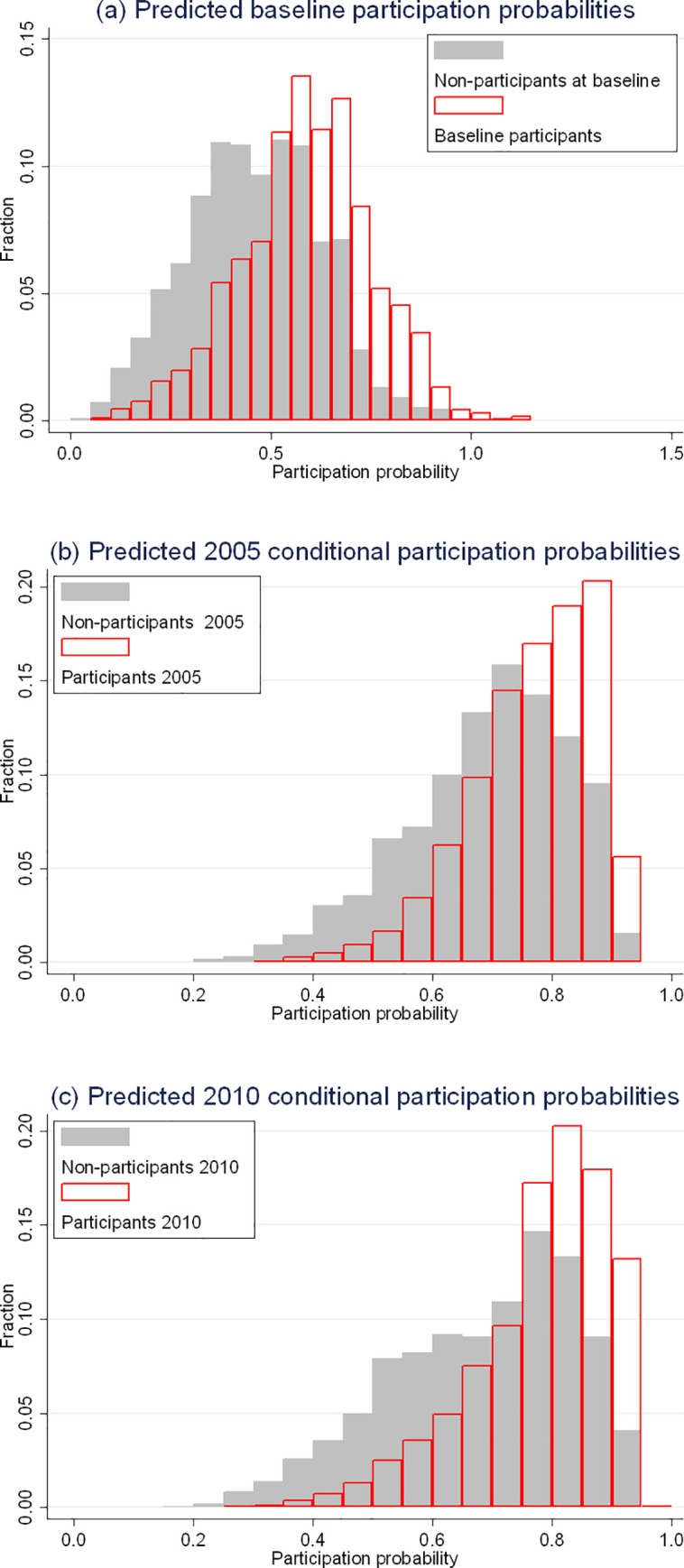
Histograms of predicted participation probabilities at baseline (a), the 2005 follow-up (b), and the 2010 follow-up (c). Predictions were obtained from background characteristics (see Table 1 as well as Tables A1 and A2 in S1 File) and are plotted separately for participants and non-participants of the survey in question. Non-participants in panels (b) and (c) only include individuals who had participated in the previous round(s). The bar width was set to 0.05.

Fig 1 shows histograms of predicted participation probabilities, separately for participants and non-participants in the different surveys. First, in panel (a), we display the probabilities of baseline participation, as obtained from logistic regressions of participation on background variables. Although there is a substantial overlap, the central tendency is clearly higher in participants. The ability of the participation model to predict baseline participation was moderate; the AUC based on the data represented in panel (a) was 71.3%.

Similarly, in panel (b), we display the corresponding histogram of predicted probabilities of participation in the 2005 follow-up, distinguishing between baseline participants who participated in the 2005 follow-up and baseline participants who did not. Finally, in panel (c), we display a histogram of predicted probabilities of participation in the 2010 follow-up, distinguishing between individuals who participated in all the three survey rounds and those only participated in the first two. Again, there were substantial overlaps but clear differences in central tendency. The AUCs were 67.1 and 70.1%.

Table 1 displays relative frequencies of the background characteristics that were used to estimate the participation probabilities at baseline. The characteristics are displayed separately for the target population, the baseline cohort, for individuals who additionally participated in the 2005 follow-up, and for individuals who in addition to this also participated in the 2010 follow-up. Compared to the target population, participants in each participant cohort were somewhat less likely to belong to the youngest age group (aged 18–40 in 1999) and were much less likely to have been hospitalized for the diseases considered. They were also somewhat more likely to be female, born in Sweden, married, high-educated, employed, and to have higher income. The discrepancies with the target population grew stronger for the 2005 follow-up, and even stronger for the 2010 follow-up. Individuals in the oldest age group were overrepresented at baseline and in the 2005 follow-up but underrepresented in 2010.

**Table 1:** Background characteristics that were used to estimate baseline participation probability.

	Target population (n = 60,000)	SPHC 99/00 (baseline) (n = 12,432)	SPHC 2005 (n = 9,315)	SPHC 2010 (n = 7,104)	Reweighted SPHC 99/00	Reweighted SPHC 2005	Reweighted SPHC 2010
*Socio-demographics at baseline (end of 1999)*							
Age							
18–40	38.4	35.2	33.1	32.3	38.3	38.5	38.9
41–59	37.2	38.6	41.8	45.9	37.2	37.5	37.8
60–80	24.4	26.2	25.2	21.7	24.5	24.0	23.3
Female	50.1	53.5	54.8	55.1	49.8	49.9	50.4
Country of birth							
Sweden	86.4	89.7	91.0	91.6	86.4	86.3	85.7
Nordic	3.0	2.3	2.3	2.2	3.0	3.0	3.2
European	6.8	5.3	4.5	4.1	6.8	6.7	6.7
Other	2.3	1.8	1.6	1.5	2.3	2.1	2.1
Married	50.2	55.3	57.6	59.0	50.4	50.3	49.9
Education							
Primary	32.5	27.5	25.2	22.9	32.4	34.4	36.4
Secondary	43.1	43.8	43.8	43.7	43.2	41.7	40.2
Tertiary	24.4	28.7	31.0	33.4	24.4	23.9	23.4
Employment status							
Employed	63.5	66.6	69.3	73.2	63.5	64.9	66.9
Unemployed	11.4	8.7	7.9	7.7	11.3	10.9	11.1
Sickness	2.8	2.8	2.7	2.7	3.0	3.1	2.8
Retired	22.2	21.9	20.1	16.4	22.2	21.2	19.3
Disposable income							
Quartile 1	25.1	20.9	18.8	16.7	24.9	23.8	22.3
Quartile 2	25.0	23.5	22.2	21.1	25.1	25.1	25.1
Quartile 3	25.0	26.6	28.0	28.8	25.1	26.1	27.1
Quartile 4	25.0	29.0	31.0	33.4	25.0	24.9	25.4
*Disease history at baseline (1987–1999)*							
Circulatory	7.1	3.9	3.5	2.7	7.8	7.3	5.8
Diabetes	0.8	0.4	0.4	0.2	1.0	1.0	0.6
Neoplasms	4.2	2.6	2.6	2.5	4.8	4.8	4.7
Respiratory	4.6	2.3	2.2	2.1	5.3	5.5	5.7
Digestive	7.5	4.1	4.0	3.8	8.4	8.3	8.0
Mental	3.6	1.3	1.0	0.8	3.9	4.0	3.9

Numbers (%) are reported separately for the target population, for individuals who participated in the baseline survey, for individuals who also participated in the 2005 follow-up, and for individuals who additionally participated in the 2010 follow-up. The applied reweightings aimed to obtain resemblance with the target population. For the baseline 99/00 cohort, simple reweighting was applied, based on the above characteristics that were used to predict baseline participation. For the 2005 cohort, double reweighting was applied, also based on the characteristics in Table A1 in S1 File, which were used to predict participation in the 2005 cohort given participation at baseline. Further, for the 2010 cohort, triple reweighting was applied, additionally based on the characteristics in Table A2 in S1 File, which were used to predict participation in the 2010 cohort given participation in the 2005 cohort.

In a similar fashion to Table 1, Tables A1 and A2 in S1 File provide relative frequencies for the characteristics that were used to estimate participation probabilities in the 2005 and 2010 follow-ups. Here, the target population is not displayed, as not everyone in the target population was alive or lived in Sweden post-baseline, and we instead compare characteristics across participants in the different survey rounds. Apart from confirming the patterns in Table 1, the results show that participants in later follow-ups were more likely to report better health, less likely to have a sedentary lifestyle, less likely to smoke, and less likely to be overweight. However, the observed differences were rather small.

Table 1, Tables A1 and A2 in S1 File also report descriptive statistics after reweighting the participant samples. Specifically, in Table 1, the three participant cohorts were reweighted (with simple, double, or triple reweighting) to obtain resemblance with the target population. Overall, this successfully produced descriptives close to those in the target population. In Tables A1 and A2 in S1 File, (simple or double) reweightings were applied to obtain resemblance with the baseline or 2005 follow-ups, also producing satisfactory results.

The share of individuals reporting bad health was 29% at baseline, 31% at the first follow-up, and 30% at the second follow-up. In Table 2, we display odds ratios (ORs) for multivariable associations between bad self-assessed health and explanatory variables, obtained from logistic regressions. Women, foreign-born, and older individuals were more likely to report bad health. In unweighted models at baseline, for example, the odds ratios were 1.35 for female, 1.36 for foreign-born, and 1.95 for the oldest age group when compared to the youngest. Individuals were also more likely to report bad health if they had health problems as measured by previous hospital visits, particularly mental health (odds ratio of 2.07) or if they had a sedentary lifestyle (2.21), smoked daily (1.41) or were overweight (1.28). In contrast, higher-educated and married individuals were less likely to report bad health, with odds ratios of 0.57 for tertiary education compared to primary education and 0.81 for married. The patterns in the follow-up surveys were similar to those in the baseline data. In the models estimated on follow-up data, we also included bad overall self-assessed health from the last survey as a covariate, which unsurprisingly produced large odds ratios, amounting to 5.38 in the first follow-up and 6.31 in the second. Estimates from univariable models (Table A3 in S1 File) were rather similar to the multivariable ones. Throughout all the results, almost all estimates turned out statistically significant at the 5% level, the estimate for married being the main exception (insignificant in the multivariable model at baseline and in both the univariable and multivariable models in the first follow-up). (Note that, since multivariable models applied to the follow-up surveys were adjusted for previous bad health, estimates for variables other than previous bad health represent effects on *changes* in health.)

**Table 2:** Unweighted and reweighted associations (multivariable).

	Unweighted OR (95% CI)	Weighted OR (95% CI)
**Panel A: Outcome = Bad self-assessed health in 1999/2000 (n = 12,432, of which 3,655 “bad” and 8,777 “not bad”)**		
(Socio-demographic explanatory variables are defined in 1999; diseases 1987–1999; self-assessed variables come from the 2000 survey)		
Age		
18–40	1.00 (Ref.)	1.00 (Ref.)
41–59	1.64 (1.48–1.82)	1.68 (1.50–1.89)
60–80	1.95 (1.74–2.19)	2.07 (1.82–2.35)
Female	1.35 (1.25–1.47)	1.35 (1.23–1.48)
Foreign-born	1.36 (1.19–1.54)	1.31 (1.14–1.51)
Married	0.81 (0.74–0.88)	0.82 (0.74–0.90)
Education		
Primary	1.00 (Ref.)	1.00 (Ref.)
Secondary	0.75 (0.68–0.82)	0.77 (0.69–0.85)
Tertiary	0.57 (0.51–0.64)	0.59 (0.52–0.67)
Hospitalized for circulatory conditions	1.80 (1.48–2.19)	1.65 (1.32–2.06)
Hospitalized for diabetes	1.49 (0.81–2.74)	1.17 (0.50–2.74)
Hospitalized for neoplasms	1.54 (1.22–1.95)	1.40 (1.05–1.87)
Hospitalized for respiratory conditions	1.35 (1.04–1.74)	1.25 (0.92–1.69)
Hospitalized for digestive conditions	1.66 (1.37–2.01)	1.65 (1.34–2.04)
Hospitalized for mental conditions	2.07 (1.49–2.88)	1.75 (1.20–2.56)
Sedentary lifestyle at baseline	2.21 (1.99–2.46)	2.29 (2.04–2.58)
Daily smoking at baseline	1.41 (1.27–1.56)	1.39 (1.24–1.56)
Overweight at baseline	1.28 (1.18–1.39)	1.26 (1.15–1.39)
**Panel B: Outcome = Bad self-assessed health in 2005 (n = 9,315, of which 2,883 “bad” and 6,432 “not bad”)**		
(Socio-demographic explanatory variables are defined in 2004; diseases 1987–2004; self-assessed variables come from the 2000 survey)		
Age		
23–45	1.00 (Ref.)	1.00 (Ref.)
46–64	1.11 (0.98–1.26)	1.21 (1.04–1.41)
65–85	1.47 (1.27–1.70)	1.65 (1.39–1.95)
Female	1.16 (1.05–1.28)	1.15 (1.03–1.30)
Foreign-born	1.39 (1.18–1.64)	1.27 (1.04–1.55)
Married	0.96 (0.86–1.06)	0.96 (0.85–1.09)
Education		
Primary	1.00 (Ref.)	1.00 (Ref.)
Secondary	0.93 (0.82–1.06)	0.95 (0.82–1.11)
Tertiary	0.64 (0.55–0.73)	0.69 (0.58–0.81)
Hospitalized for circulatory conditions	1.52 (1.35–1.72)	1.41 (1.22–1.65)
Hospitalized for diabetes	1.36 (0.98–1.87)	1.44 (0.93–2.24)
Hospitalized for neoplasms	1.02 (0.88–1.20)	1.05 (0.86–1.27)
Hospitalized for respiratory conditions	1.34 (1.11–1.60)	1.46 (1.15–1.85)
Hospitalized for digestive conditions	1.08 (0.94–1.25)	1.18 (0.99–1.41)
Hospitalized for mental conditions	1.53 (1.20–1.95)	1.77 (1.29–2.44)
Bad self-assessed health at baseline	5.38 (4.85–5.97)	5.17 (4.57–5.84)
Sedentary lifestyle at baseline	1.39 (1.21–1.60)	1.37 (1.15–1.62)
Daily smoking at baseline	1.32 (1.16–1.51)	1.32 (1.13–1.54)
Overweight at baseline	1.31 (1.19–1.45)	1.40 (1.25–1.58)
**Panel C: Outcome = Bad self-assessed health in 2010 (n = 7,104, of which 2,112 “bad” and 4,992 “not bad”)**		
(Socio-demographic explanatory variables are defined in 2009; diseases 1987–2009; self-assessed variables come from the 2005 survey)		
Age		
28–50	1.00 (Ref.)	1.00 (Ref.)
51–69	1.05 (0.90–1.21)	1.12 (0.92–1.36)
70–90	1.93 (1.62–2.30)	1.85 (1.44–2.38)
Female	1.16 (1.03–1.31)	1.17 (1.00–1.37)
Foreign-born	1.24 (1.01–1.52)	1.21 (0.92–1.58)
Married	0.87 (0.77–0.98)	0.81 (0.69–0.95)
Education		
Primary	1.00 (Ref.)	1.00 (Ref.)
Secondary	0.87 (0.74–1.02)	0.80 (0.65–0.98)
Tertiary	0.69 (0.58–0.81)	0.62 (0.50–0.77)
Hospitalized for circulatory conditions	1.20 (1.03–1.39)	1.18 (0.98–1.42)
Hospitalized for diabetes	1.78 (1.29–2.47)	1.97 (1.36–2.84)
Hospitalized for neoplasms	1.17 (1.01–1.35)	1.15 (0.95–1.39)
Hospitalized for respiratory conditions	1.21 (1.03–1.42)	1.27 (1.02–1.60)
Hospitalized for digestive conditions	1.18 (1.03–1.35)	1.33 (1.11–1.60)
Hospitalized for mental conditions	1.77 (1.41–2.21)	1.97 (1.39–2.78)
Bad health at first follow-up	6.31 (5.59–7.13)	5.72 (4.86–6.72)
Sedentary lifestyle at first follow-up	1.49 (1.23–1.80)	1.56 (1.22–1.99)
Daily smoking at first follow-up	1.38 (1.16–1.64)	1.29 (1.04–1.61)
Overweight at first follow-up	1.44 (1.27–1.62)	1.34 (1.15–1.58)

The table shows odds ratios from logistic regressions for very poor/poor/fair self-assessed health versus the listed socio-demographic, disease, and self-assessed variables. Reference groups for binary variables are omitted. The reweightings aimed to obtain resemblance with the target population.

Reweighting the participant samples to resemble the target population had generally small or moderate effects on the associations. The estimates for age tended to grow somewhat larger, for example the effect of the highest age group grew from an OR of 1.95 to 2.07 in the multivariable model and from 2.17 to 2.44 in the univariable model when applied to the baseline cohort. Other estimates, such as for mental health in the baseline cohort, reduced in size. Roughly half of the ORs, 55 out of 100, became more extreme compared to 1 upon reweighting (21 of 50 of those from the multivariable models and 34 of 50 of those from the univariable models). There were 44 estimates that moved closer to 1 without any change in the direction of the effect, and only one estimate changed direction; however, this one (the estimate for married in the univariable model in the baseline cohort) was close to 1 and statistically insignificant at the 5% level both with and without reweighting.

**Fig 2:**
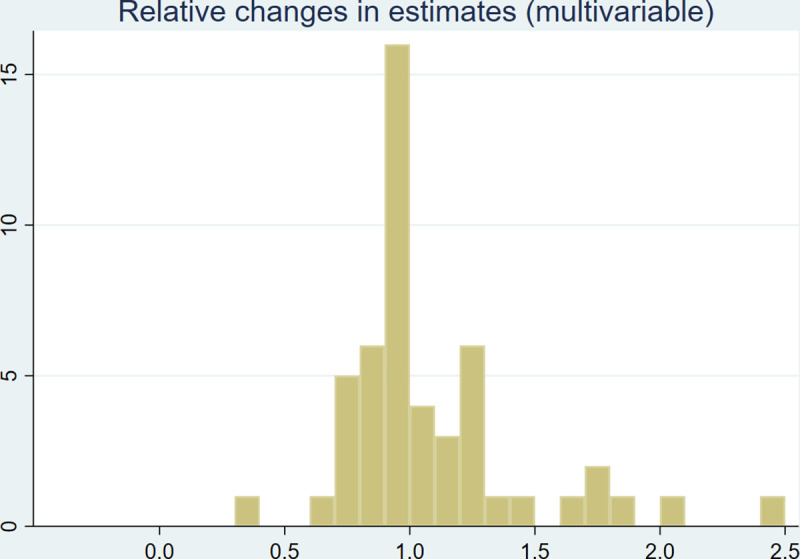
Histogram of relative changes in (multivariable) associations. The figure shows ratios of log odds ratios from reweighted and unweighted multivariable models. The bar width was set to 0.1.

In Fig 2, we provide a histogram of relative changes in estimates from the multivariable models upon reweighting. Relative changes were calculated based on B-coefficients (i.e., log(OR)), dividing the estimate from the weighted model by the estimate from the unweighted one. A corresponding figure for estimates from univariable models is shown in Fig A1 in S1 File. Among the 50 estimates from the multivariable models, 20 moved by less than 10% in either direction upon reweighting whereas 29 moved by less than 20%. The univariable estimates exhibited somewhat less variability, with 23 out of 50 estimates moving by less than 10% and 40 moving by less than 20%.

## Discussion

In this investigation, we examined selective participation at baseline and in first two follow-ups of the SPHC study and examined the ability of sample reweighting to improve the validity of various associations with self-assessed health. The variables used in the reweightings represented a broad range of characteristics and were based on both register-based information such as socio-demographics and disease history, and information on health and health-related behaviors from previous rounds of the survey. In summary, we found that the consequences of reweighting varied across the different associations considered. Few systematic patterns could be detected, although the estimates for (high) age generally got stronger in reweighted models, suggesting an underestimate in models not accounting for selective participation. Typically, the consequences of selective participation were modest, often amounting to a change of less than 10% of the B-coefficient, and in most cases less than 20%. The qualitative conclusions hardly ever changed.

The limited impact of reweighting could be interpreted to suggest that the SPHC cohort is quite representative of the target population, in the sense that the same associations would have been present if one were able to estimate the models on the full population. However, it is still possible that there were some unobserved determinants of participation that we were unable to account for, which could lead both our unweighted and reweighted results to be biased. In a previous study based on the MDC (Malmö Diet and Cancer) cohort, we found that mortality was markedly different across participants and the full population, and that reweighting on observed register-based variables helped only little to mitigate the biases [15]. On the other hand, associations between mortality and various register-based variables were similar across the full population and the participants already without reweighting, suggesting that associations may be rather insensitive to selective participation. This has also been implied by several other previous studies [1, 14, 20] including one on SPHC [18], which compared a number of associations with mortality as well as drug purchases across baseline and follow-up participants. Partly different conclusions, however, have been obtained by recent investigations of the UK Biobank, a cohort with a response rate as low as 5.5%. In this cohort, certain associations with CVD mortality were found to be substantially different from the corresponding ones in the more representative Health Survey for England/Scottish Health Surveys (HSE-SHS) [21], and reweighting the biobank had substantial impacts on some of the associations [16]. Overall, we conclude that cohort representativeness and the need for reweighting should be decided on a case-by-case basis.

Selection bias can arise for different reasons. Some epidemiologists use the term selection bias only when referring to collider stratification bias, i.e., the bias that can arise when selection into a study is related to both exposure and outcome [6, 7]. However, a broader and for our purposes more relevant definition states that “Selection bias arises when the estimate of occurrence or etiologic effect obtained from a study differs systematically from what would have been obtained had information from the source population been available” [5]. Such systematic differences between an estimate from a select sample and the estimate that would have prevailed in the full population can arise not only due to selection on colliders, but also if the causal effect of an exposure varies depending on some variable whose prevalence is different in participants and the population at large, i.e., in the presence of effect heterogeneity [3, 8].

**Fig 3:**
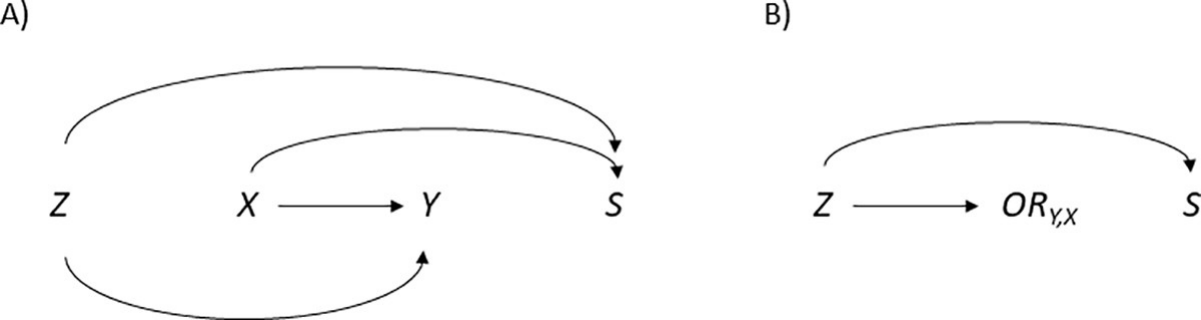
Directed acyclic graphs (DAGs) showing how selection bias can arise. Panel A displays an example of collider bias (where estimates from the sample lack causal interpretation), whereas panel B displays an example of causal effect heterogeneity (where the effects are inherently different across participants and the full population).

Fig 3 provides two stylized examples of how selection bias can arise, illustrated with directed acyclic graphs (DAGs) [22]. First, in panel A, we show a typical collider bias scenario. *S* represents participation in the sample (either at baseline or in a follow-up), *Y* is the outcome self-assessed bad health, and *X* and *Z* are two potential determinants of both the outcome and sample participation, such as age and healthy lifestyle. When an analysis is restricted to participants (*S* = 1), a non-causal statistical association between variables that point to *S* (here: *X* and *Z*) will generally arise, in turn distorting the association between *X* and *Y* (unless *Z* is being adjusted for in a regression, in which case the bias may at least partly be removed) [2, 6].

Second, in panel B, we show an example of effect heterogeneity, using a DAG variant that includes the size of the association between *Y* and *X* (*OR*_*Y*,*X*_) as a node [23]. The size of the association (*OR*_*Y*,*X*_) is assumed to depend on *Z*, which also influences sample participation. Compared to the population, participants will tend to have different values on *Z*, and as a result, the odds ratios will differ, implying a selection bias. (Regression adjustment for *Z* does not help to mitigate this type of selection bias.) Reweighing with respect to *Z* has the potential to eliminate selection bias, both in this scenario and in the scenario depicted in panel A [2, 6, 11].

Taking our results for (high) age, the larger effects that were generally obtained upon reweighting could reflect that sample participation is related to age as well as other variables, causing a (downward) collider bias in the unweighted estimates. However, it could also reflect that the effect of age is heterogeneous with respect to some background variable, where the effects of high age are smaller among participants than in the population at large.

We believe that the investigation presented in this article should become a standard procedure that researchers apply to assess and improve the validity of cohorts, whenever possible. Applying the framework is relatively straightforward, and even if the reweighting turns out to make little difference, it provides a source of comfort to know that selective participation (at least with respect to observed factors) does not pose a substantial threat to validity. The framework is particularly easy to apply in a Nordic context, given the extensive availability of data from government registers, which can often be combined with health cohorts. As demonstrated, however, it is not necessary to have any direct links between the sample and the full population data, i.e., a completely separate set of data from a representative population can be used, provided that it contains variables that are also available in the sample dataset. This feature allows for meaningful applications in a wide range of contexts, also where the availability of population-level data is somewhat more limited. Along with existing methods to assess and improve the validity of cohorts, such as adjustments for confounding and evaluations of the quality of data, reweighting for representativity should constitute a helpful addition to the epidemiologic toolbox.

## Supporting information

S1 File Table A1: Background characteristics that were used to estimate participation probability in the 2005 follow-up; Table A2: Background characteristics that were used to estimate participation probability in the 2010 follow-up; Table A3: Unweighted and reweighted univariable associations; Fig A1. Histogram of relative changes in univariable associations.
(DOCX)
