# Supplementary material for: Reweighting a Swedish health questionnaire survey using extensive population register and self-reported data for assessing and improving the validity of longitudinal associations
Source: PLoS One. 2021 Jul 1;16(7):e0253969. doi: 10.1371/journal.pone.0253969 (PMC8248630; doi:10.1371/journal.pone.0253969)
Supplement: S1 File — Histogram of relative changes in univariable associations. (DOCX) [file pone.0253969.s001.docx]

**S1 File**

**Table A1: Background characteristics that were used to estimate participation probability in the 2005 follow-up**

|  | 99/00 cohort (n=12,432) | 2005 cohort (n=9,315) | 2010 cohort (n=7,104) | Reweighted 2005 cohort | Reweighted 2010 cohort |
| --- | --- | --- | --- | --- | --- |
| *Socio-demographics at the first follow-up (end of 2004, or latest available)* | | | | | |
| Age |  |  |  |  |  |
| 23-45 | 35.2 | 33.1 | 32.3 | 35.3 | 35.3 |
| 46-64 | 38.6 | 41.8 | 45.9 | 38.8 | 38.9 |
| 65-85 | 26.2 | 25.2 | 21.7 | 26.0 | 25.9 |
| Female | 53.5 | 54.8 | 55.1 | 53.6 | 53.8 |
| Country of birth |  |  |  |  |  |
| Sweden | 89.7 | 91.0 | 91.6 | 89.6 | 89.3 |
| Nordic | 2.3 | 2.3 | 2.2 | 2.3 | 2.4 |
| European | 5.3 | 4.5 | 4.1 | 5.3 | 5.4 |
| Other | 1.8 | 1.6 | 1.5 | 1.7 | 1.7 |
| Married | 55.3 | 57.6 | 58.9 | 55.4 | 55.3 |
| Education |  |  |  |  |  |
| Primary | 26.1 | 22.0 | 19.9 | 25.8 | 28.3 |
| Secondary | 41.3 | 42.5 | 41.8 | 41.5 | 39.8 |
| Tertiary | 32.6 | 35.4 | 38.3 | 32.7 | 32.0 |
| Employment status |  |  |  |  |  |
| Employed | 59.8 | 62.0 | 65.9 | 60.0 | 60.3 |
| Unemployed | 6.1 | 5.3 | 5.3 | 6.2 | 6.5 |
| Sickness | 3.2 | 2.9 | 2.8 | 3.2 | 3.1 |
| Retired | 30.8 | 29.7 | 26.0 | 30.6 | 30.1 |
| Disposable income |  |  |  |  |  |
| Quartile 1 | 22.4 | 20.5 | 17.9 | 22.4 | 22.0 |
| Quartile 2 | 24.1 | 23.2 | 22.1 | 24.0 | 23.9 |
| Quartile 3 | 25.8 | 26.6 | 27.5 | 25.8 | 25.9 |
| Quartile 4 | 27.7 | 29.7 | 32.5 | 27.7 | 28.1 |
| *Disease history at the first follow-up (1987-2004)* | | | | | |
| Circulatory | 41.4 | 40.1 | 38.2 | 41.4 | 41.2 |
| Diabetes | 2.3 | 2.1 | 1.8 | 2.3 | 2.3 |
| Neoplasms | 13.7 | 12.6 | 12.2 | 13.5 | 14.1 |
| Respiratory | 8.3 | 7.5 | 7.1 | 8.3 | 8.4 |
| Digestive | 14.9 | 14.9 | 14.2 | 15.2 | 15.2 |
| Mental | 4.8 | 3.9 | 3.1 | 4.9 | 4.8 |
| *Self-assessed variables in the baseline survey (1999/2000)* | | | | | |
| Self-assessed health |  |  |  |  |  |
| Very good | 19.2 | 20.2 | 21.2 | 19.2 | 19.0 |
| Good | 51.4 | 52.3 | 53.2 | 51.5 | 51.4 |
| Fair | 23.6 | 22.5 | 21.0 | 23.7 | 23.8 |
| Poor | 5.0 | 4.4 | 4.2 | 4.9 | 5.1 |
| Very poor | 0.8 | 0.6 | 0.5 | 0.8 | 0.7 |
| Physical activity |  |  |  |  |  |
| Sedentary | 14.9 | 13.4 | 12.5 | 14.9 | 14.9 |
| Moderate | 63.0 | 63.8 | 63.4 | 63.0 | 63.2 |
| Regular | 19.5 | 20.5 | 22.1 | 19.5 | 19.6 |
| Intense | 2.6 | 2.3 | 2.1 | 2.7 | 2.3 |
| Smoking |  |  |  |  |  |
| Daily | 18.5 | 17.1 | 16.0 | 18.6 | 19.1 |
| Not daily | 4.4 | 4.3 | 4.3 | 4.3 | 4.4 |
| Not smoking | 77.1 | 78.6 | 79.7 | 77.0 | 76.6 |
| Overweight | 44.5 | 44.4 | 43.6 | 44.6 | 44.6 |

Numbers (%) are reported separately for all baseline participants, individuals who also participated in the 2005 follow-up, and individuals who additionally participated in the 2010 follow-up. The applied reweightings aimed to obtain resemblance with baseline participants. For the 2005 cohort, simple reweighting was applied, based on the above characteristics, which were used to predict participation in the 2005 cohort given baseline participation. For the 2010 cohort, double reweighting was applied, additionally based on the characteristics in Table A2, which were used to predict participation in the 2010 cohort given participation in the 2005 cohort.

**Table A2: Background characteristics that were used to estimate participation probability in the 2010 follow-up**

|  | 2005 cohort (n=9,315) | 2010 cohort (n=7,104) | Reweighted 2010 cohort |
| --- | --- | --- | --- |
| *Socio-demographics at the first follow-up (end of 2009, or latest available)* | | | |
| Age |  |  |  |
| 28-50 | 33.1 | 32.3 | 35.3 |
| 51-69 | 41.8 | 45.9 | 38.9 |
| 70-90 | 25.2 | 21.7 | 25.9 |
| Female | 54.8 | 55.1 | 53.8 |
| Country of birth |  |  |  |
| Sweden | 91.0 | 91.6 | 89.3 |
| Nordic | 2.3 | 2.2 | 2.4 |
| European | 4.5 | 4.1 | 5.4 |
| Other | 1.6 | 1.5 | 1.7 |
| Married | 57.6 | 58.9 | 55.3 |
| Education |  |  |  |
| Primary | 24.3 | 19.8 | 28.0 |
| Secondary | 40.2 | 41.1 | 39.2 |
| Tertiary | 35.6 | 39.1 | 32.8 |
| Employment status |  |  |  |
| Employed | 55.4 | 58.2 | 54.3 |
| Unemployed | 4.3 | 4.0 | 4.7 |
| Sickness | 1.2 | 1.1 | 1.3 |
| Retired | 39.2 | 36.7 | 39.7 |
| Disposable income |  |  |  |
| Quartile 1 | 21.7 | 19.1 | 23.6 |
| Quintile 2 | 25.1 | 24.4 | 25.5 |
| Quintile 3 | 26.0 | 27.3 | 25.6 |
| Quintile 4 | 27.1 | 29.3 | 25.4 |
| *Disease history at the first follow-up (1987-2009)* | | | |
| Circulatory | 63.3 | 61.4 | 64.2 |
| Diabetes | 3.4 | 2.9 | 3.6 |
| Neoplasms | 24.1 | 22.4 | 24.9 |
| Respiratory | 15.6 | 14.4 | 16.2 |
| Digestive | 27.5 | 26.5 | 28.2 |
| Mental | 8.0 | 6.5 | 9.2 |
| *Self-assessed variables in the 2005 survey* | | | |
| Self-assessed health |  |  |  |
| Very good | 18.0 | 19.6 | 17.2 |
| Good | 51.0 | 52.4 | 50.2 |
| Fair | 25.7 | 23.5 | 26.7 |
| Poor | 4.6 | 4.0 | 5.0 |
| Very poor | 0.7 | 0.5 | 0.8 |
| Physical activity |  |  |  |
| Sedentary | 11.1 | 9.3 | 12.1 |
| Moderate | 65.9 | 65.9 | 65.9 |
| Regular | 21.3 | 23.2 | 20.3 |
| Intense | 1.6 | 1.5 | 1.7 |
| Smoking |  |  |  |
| Daily | 13.4 | 12.1 | 14.8 |
| Not daily | 3.0 | 2.8 | 3.0 |
| Not smoking | 83.7 | 85.1 | 82.2 |
| Overweight | 48.7 | 48.4 | 49.2 |

Numbers (%) are reported separately for all participants in the 2005 follow-up and individuals who also participated in the 2010 follow-up. The applied reweighting aimed to obtain resemblance with 2005 participants. Simple reweighting was applied based on the above characteristics, which were used to predict participation in the 2010 cohort given participation in the 2005 cohort.

**Table A3: Unweighted and reweighted univariable associations.**

|  | Unweighted  OR (95% CI) | Weighted  OR (95% CI) |
| --- | --- | --- |
| **Panel A: Outcome = Bad self-assessed health in 1999/2000 (n=12,432, of which 29% were “bad”)**  (Socio-demographic explanatory variables are defined in 1999; diseases 1987-1999; self-assessed variables come from the 2000 survey) | | |
| Age |  |  |
| 18-40 | 1.00 (Ref.) | 1.00 (Ref.) |
| 41-59 | 1.61 (1.47-1.77) | 1.71 (1.54-1.90) |
| 60-80 | 2.17 (1.96-2.40) | 2.44 (2.18-2.74) |
| Female | 1.25 (1.16-1.36) | 1.19 (1.09-1.30) |
| Foreign-born | 1.43 (1.26-1.61) | 1.37 (1.20-1.56) |
| Married | 0.97 (0.89-1.04) | 1.01 (0.93-1.11) |
| Education |  |  |
| Primary | 1.00 (Ref.) | 1.00 (Ref.) |
| Secondary | 0.61 (0.56-0.67) | 0.62 (0.56-0.68) |
| Tertiary | 0.43 (0.39-0.48) | 0.43 (0.38-0.48) |
| Hospitalized for circulatory conditions | 2.50 (2.08-3.00) | 2.58 (2.09-3.19) |
| Hospitalized for diabetes | 2.41 (1.37-4.25) | 2.26 (1.12-4.53) |
| Hospitalized for neoplasms | 2.03 (1.63-2.53) | 2.13 (1.63-2.79) |
| Hospitalized for respiratory conditions | 1.60 (1.26-2.03) | 1.71 (1.28-2.27) |
| Hospitalized for digestive conditions | 2.15 (1.80-2.57) | 2.39 (1.93-2.94) |
| Hospitalized for mental conditions | 2.83 (2.07-3.86) | 2.56 (1.80-3.65) |
| Sedentary lifestyle at baseline | 2.35 (2.13-2.60) | 2.44 (2.17-2.74) |
| Daily smoking at baseline | 1.62 (1.48-1.79) | 1.62 (1.45-1.81) |
| Overweight at baseline | 1.44 (1.33-1.56) | 1.48 (1.36-1.62) |
| **Panel B: Outcome = Bad self-assessed health in 2005 (n=9,315, of which 2,883 “bad” and 6,432 “not bad”)**  (Socio-demographic explanatory variables are defined in 2004; diseases 1987-2004; self-assessed variables come from the 2000 survey) | | |
| Age |  |  |
| 23-45 | 1.00 (Ref.) | 1.00 (Ref.) |
| 46-64 | 1.45 (1.31-1.62) | 1.55 (1.36-1.76) |
| 65-85 | 2.09 (1.86-2.35) | 2.32 (2.00-2.68) |
| Female | 1.18 (1.08-1.29) | 1.13 (1.01-1.26) |
| Foreign-born | 1.53 (1.32-1.77) | 1.42 (1.18-1.71) |
| Married | 0.94 (0.86-1.02) | 0.96 (0.86-1.07) |
| Education |  |  |
| Primary | 1.00 (Ref.) | 1.00 (Ref.) |
| Secondary | 0.71 (0.64-0.79) | 0.68 (0.60-0.78) |
| Tertiary | 0.40 (0.36-0.46) | 0.39 (0.34-0.45) |
| Hospitalized for circulatory conditions | 2.24 (2.04-2.44) | 2.38 (2.14-2.66) |
| Hospitalized for diabetes | 2.60 (1.96-3.45) | 3.10 (2.10-4.58) |
| Hospitalized for neoplasms | 1.50 (1.32-1.70) | 1.54 (1.32-1.80) |
| Hospitalized for respiratory conditions | 1.85 (1.58-2.16) | 1.99 (1.61-2.45) |
| Hospitalized for digestive conditions | 1.72 (1.53-1.93) | 1.93 (1.66-2.24) |
| Hospitalized for mental conditions | 2.83 (2.29-3.50) | 3.02 (2.26-4.05) |
| Sedentary lifestyle at baseline | 6.63 (6.00-7.32) | 6.64 (5.89-7.48) |
| Daily smoking at baseline | 1.91 (1.69-2.16) | 1.89 (1.62-2.19) |
| Overweight at baseline | 1.67 (1.50-1.87) | 1.72 (1.50-1.99) |
| Hospitalized for circulatory conditions | 1.57 (1.44-1.72) | 1.70 (1.53-1.90) |
| **Panel C: Outcome = Bad self-assessed health in 2010 (n=7,104, of which 2,112 “bad” and 4,992 “not bad”)**  (Socio-demographic explanatory variables are defined in 2009; diseases 1987-2009; self-assessed variables come from the 2005 survey) | | |
| Age |  |  |
| 28-50 | 1.00 (Ref.) | 1.00 (Ref.) |
| 51-69 | 1.35 (1.19-1.52) | 1.37 (1.14-1.64) |
| 70-90 | 2.49 (2.16-2.86) | 2.51 (2.05-3.06) |
| Female | 1.15 (1.04-1.28) | 1.13 (0.98-1.31) |
| Foreign-born | 1.56 (1.31-1.85) | 1.58 (1.22-2.04) |
| Married | 0.80 (0.72-0.88) | 0.74 (0.64-0.86) |
| Education |  |  |
| Primary | 1.00 (Ref.) | 1.00 (Ref.) |
| Secondary | 0.70 (0.61-0.80) | 0.61 (0.51-0.73) |
| Tertiary | 0.41 (0.35-0.47) | 0.33 (0.28-0.40) |
| Hospitalized for circulatory conditions | 2.11 (1.89-2.36) | 2.37 (2.04-2.75) |
| Hospitalized for diabetes | 2.83 (2.14-3.75) | 3.35 (2.25-4.98) |
| Hospitalized for neoplasms | 1.53 (1.36-1.72) | 1.60 (1.37-1.87) |
| Hospitalized for respiratory conditions | 1.61 (1.40-1.84) | 1.84 (1.49-2.27) |
| Hospitalized for digestive conditions | 1.73 (1.55-1.94) | 2.06 (1.77-2.41) |
| Hospitalized for mental conditions | 2.64 (2.18-3.19) | 3.02 (2.26-4.03) |
| Bad health at first follow-up | 7.86 (7.00-8.82) | 7.80 (6.66-9.15) |
| Sedentary lifestyle at first follow-up | 2.27 (1.93-2.67) | 2.22 (1.79-2.76) |
| Daily smoking at first follow-up | 1.80 (1.55-2.08) | 1.79 (1.47-2.17) |
| Overweight at first follow-up | 1.69 (1.52-1.87) | 1.66 (1.44-1.91) |

The table shows odds ratios from logistic regressions for very poor/poor/fair self-assessed health versus the listed socio-demographic, disease, and self-assessed variables. Reference groups for binary variables are omitted. The reweightings aimed to obtain resemblance with the target population.


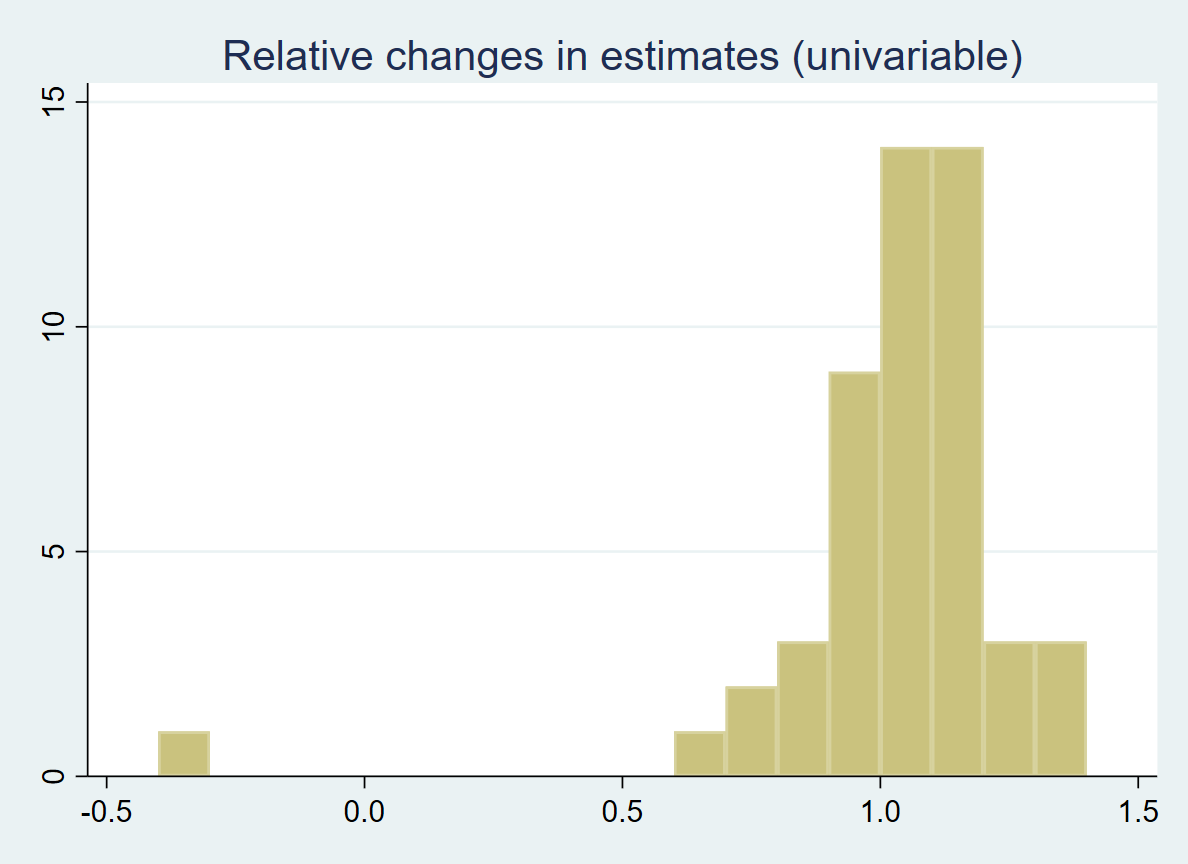


**Fig A1. Histogram of relative changes in univariable associations.** The figure shows ratios of log odds ratios from reweighted and unweighted univariable models. The bar width was set to 0.1.
